# A Novel Anti-Infective Peptide BCCY-1 With Immunomodulatory Activities

**DOI:** 10.3389/fimmu.2021.713960

**Published:** 2021-07-22

**Authors:** Jinyang Cai, Xianwei Cui, Xing Wang, Lianghui You, Chenbo Ji, Yan Cao

**Affiliations:** ^1^ Nanjing Maternity and Child Health Care Institute, Nanjing Maternity and Child Health Care Hospital, Women’s Hospital of Nanjing Medical University, Nanjing, China; ^2^ State Key Laboratory of Reproductive Medicine, Nanjing Medical University, Nanjing, China

**Keywords:** anti-infective strategy, peptide, BCCY-1, innate immunity, monocyte

## Abstract

Antibiotic resistance has been considered to be a global threat which underscores the need to develop novel anti-infective therapeutics. Modulation of innate immunity by synthetic peptides is an attractive strategy to overcome this circumstance. We recently reported that BCCY-1, a human β-casein-derived peptide displays regulatory activities on monocytes, thereby enhancing their actions in innate immune responses. However, the function of peptide BCCY-1 in host defense against infection remains unknown. In this study, we investigated the *in vivo* characteristics and effects of peptide BCCY-1 in mouse models of bacterial infection. Following intraperitoneal injection, the peptide BCCY-1 exhibited high level of cellular uptake by monocytes without obvious toxicities. Results revealed that peptide BCCY-1, but not the scrambled version, stimulated the chemokine production and monocyte recruitment *in vivo*. Treatment with BCCY-1 enhanced the pathogen clearance and protected mice against lethal infections. Because the anti-infective effects of BCCY-1 was abolished by *in vivo* depletion of monocytes/macrophages rather than lymphocytes and granulocytes, we conclude that monocytes/macrophages are key effector cells in BCCY-1-mediated anti-infective protection. Additionally, BCCY-1 lacks direct antimicrobial activity. To our knowledge, a human β-casein-derived peptide that counters infection by selective regulation of innate immunity has not been reported previously. These results suggest peptide BCCY-1 as a promising alternative approach and a valuable complement to current anti-infective strategy.

## Introduction

The discovery of antibiotics is considered the greatest achievement in treatment of infections. However, their excessive use has resulted in the emergence of drug-resistant bacteria (1). Modulation of the innate immune response, the first line of defense against infections, thereby enhancing its capacity to elimination of pathogens is an attractive way as it is consistent with antibiotic therapies but may reduce or avoid concerns of drug-resistant bacteria and has broader applications.

Although researches into the specific components in human breast milk, which lead to the notable health benefits of exclusive breastfeeding, have been ongoing for decades, there are still largely unknown about the anti-infective factors in human breast milk and how it contributes to the regulation and development of the neonatal immune function (2). It has became clearer that milk protein-derived peptides exhibit various biological activities including antimicrobial (3), opioid agonistic (4), antihypertensive (5), and immunomodulatory effects (6, 7). The human β-casein derived peptide BCCY-1 was firstly identified in our previous study of the human milk peptidome (8). We recently reported that peptide BCCY-1 has great immunomodulatory effects on monocytes *in vitro* (9), illustrating its potential in anti-infective therapy which needs to be further investigated.

In the present study, we evaluated the biosafety, biodistribution and the anti-infective effect of peptide BCCY-1 in diverse mouse models *in vivo*.

## Materials and Methods

### Peptides

The synthetic peptide BCCY-1 comprising the 112-131 aa of human β-casein (GRVMPVLKSPTIPFFDPQIP), a scrambled version of BCCY-1 (VGTKFLPVPPPDFPQSRIIM), TAMRA-labeled BCCY-1 (TAMRA-BCCY-1), were obtained from Science Peptide Biological Technology Co., Ltd (Shanghai, China). All peptides are endotoxin free and synthesized *via* solid-phase. The purity of the peptides was determined by reverse-phase high performance liquid chromatography (HPLC) and exceeded 95%. The stock solution of peptides was made in distilled water (Thermo, USA) and stored at –20°C.

### *In Vivo* Biodistribution Analysis

For *in vivo* biodistribution analysis, peptide BCCY-1 were labeled with the dye TAMRA to prepare TAMRA-BCCY-1. TAMRA-BCCY-1 (10 mg/kg) or Saline was administered to nude mice intraperitoneally. The *in vivo* biodistribution was determined at 1 h, 3 h, 6 h, 9 h post-injection by the whole-body and *ex vivo* organ fluorescence imaging. Fluorescence imaging (λex/λem at 570 nm/620 nm, epi-illumination mode) was conducted using IVIS Spectrum imaging system (PerkinElmer). Average radiant efficiency was used to calculate the biodistribution of TAMRA-BCCY-1. The biodistribution of TAMRA-BCCY-1 in peripheral blood cells was analyzed using the BD FACSAria™ Fusion flow cytometer (BD Biosciences, USA). The FACS gating strategy is shown as **Supplementary Figure 1**.

### Animal

8-week old SPF C57BL6/J male mice were treated with BCCY-1 (10 mg/kg in saline solution) by intraperitoneal injection. Sex and age-matched mice treated with Saline or Scrambled peptides served as the control. This study was approved by the Institutional Review Board at Women’s Hospital of Nanjing Medical University, Nanjing Maternity and Child Health Care Hospital ([2017]15). After 24 or 48 h, peritoneal lavage of each mice was collected and cells in peritoneal lavage fluid were isolated *via* centrifugation. Peritoneal lavage cells were cytospun, air dried and stained using Wright-Giemsa staining kit (Nanjing Jiancheng Bioengineering Institute, China) according to the manufacturer’s instruction. The number of peritoneal lavage cells of each mice were counted. Peritoneal lavage cells were stained with F4/80-FITC and Ly-6C-PE conjugated antibodies (ebioscience, USA) for 30 min on ice. Samples were analyzed using flow cytometer (BD Biosciences, USA). The FACS gating strategy is shown as **Supplementary Figure 2**. In addition, the stained cells were cytospun, air dried, fixed with 4% polyformaldehyde and mounted in ProLong™ Gold Antifade Mountant with DAPI (Thermo, USA). The samples were analyzed with a confocal microscope (Zeiss, Germany). For murine systemic infection model, C57BL6/J mice were treated with BCCY-1 (10 mg/kg in saline solution) once per day for two days. Mid-log-phase *E.coli* ATCC 8739 were diluted to 2×10^7^~1×10^8^ colony-forming units (CFUs)/mouse in PBS. Mice were challenged by *i.p.* injection with the bacteria suspended in PBS. Mortality was recorded daily during the following 8 days. To measure bacterial CFUs, the mice were sacrificed 24 h post bacterial challenge and the peritoneal lavage fluid was obtained. Samples were spread on appropriate agar plates to count colonies. For acute toxicity studies, mice were observed for 7 days after *i.p.* injection of BCCY-1. Observations included movement, body weight and food intake. The daily food intake was measured according to the reported method (10). Daily food intake per mouse was recorded. Briefly, food placed in each cage was weighed at 9:00 am. At the same time the following day, the remaining food was weighed again. The difference in weight of the food represented daily food intake. Mice were sacrificed at day 8. Blood samples were collected and was analyzed using a blood biochemical autoanalyzer (Mindray, BC-2800vet, China). Lung, spleen, liver, kidney and heart were collected and embedded in paraffin. The histological alterations of tissue sections were assessed using hematoxylin and eosin (H&E) staining *via* microscopy. For the clodronate model, monocytes/macrophages were depleted in C57BL6/J mice by treatment with clodronate liposomes (YEASEN Biotech Co., Ltd, China) according to the manufacturer’s instruction as previously described (11). Then 2×10^7^~1×10^8^ CFUs/mouse *E.coli* ATCC 8739 were given 48 h after BCCY-1 (10 mg/kg/day, for 2 days) or a saline treatment. Nucleated cell number and bacterial counts in the peritoneal lavage fluid were determined 24 h after the initiation of infection. For the neutropenic model, mice were injected with two doses of cyclophosphamide (Cy, 200 mg/kg and 100 mg/kg) as previously described (11), and subsequently treated with BCCY-1 (10 mg/kg/day, for 2 days) and then infected as described for the murine systemic infection model. For the lymphopenia model, Rag2^-/-^ mice were used (*Rag2^tm1.1Cgn^*, Stock No: 008449, The Jackson Laboratory, USA) and subsequently treated and infected as described for the murine systemic infection model. The experimental flow charts of the different mouse models are shown as **Supplementary Figure 3**.

### Determination of MCP-1 Levels by ELISA

The peritoneal lavage fluids were collected and centrifuged at 1,000 × g for 10 min to obtain cell-free samples and stored at −80°C. Enzyme-linked immunosorbent assay (ELISA) was used to determine the concentrations of MCP-1 (CUSABIO BIOTECH CO., Ltd, China) according to manufacturer’s instructions.

### *In Vitro* Antimicrobial Assay

Briefly, serial diluted (1-250 μg/mL) aliquots of the peptide BCCY-1 were added to sterile 96-well plates in a volume of 10 μL, followed by the addition of 90 μL of diluted bacteria (2×10^5^ CFUs/mL; exponential phase). After incubation for 18-20 h at 37°C, the absorbance at 600 nm was measured using a microplate reader (Synergy H4, BioTek, USA).

### Statistical Analysis

GraphPad Prism 5/7 software (GraphPad, USA) was used for statistical data analysis, with a two-tailed Student’s t test used for comparisons. The statistical significances were calculated as p values, and p < 0.05 denoted statistical significance.

## Results

### The Biosafety of BCCY-1 *In Vivo*

**Figure 1:**
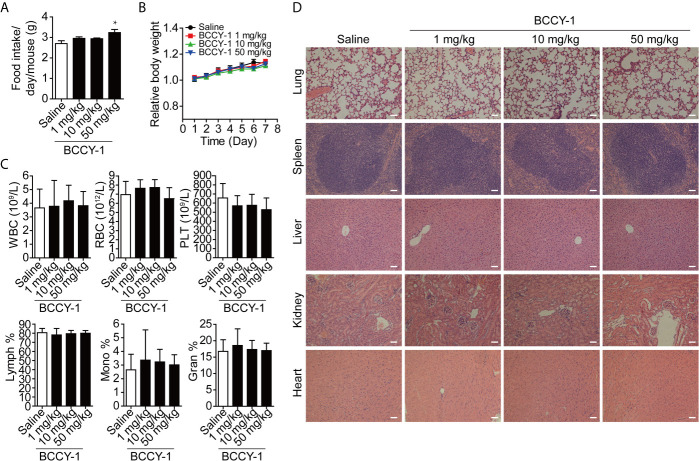
Effects of BCCY-1 on organ toxicity. **(A)** food intake/day/mouse and **(B)** body weight were monitored daily after *i.p.* injection of peptide BCCY-1 at indicated concentration. Results are expressed as the body weight relative to the initial body weight. *p < 0.05. **(C)** Routine blood analysis was performed after BCCY-1 treatment. WBC, White blood cell; RBC, red blood cell; PLT, platelet; Lymph, lymphocyte; Mono, monocyte; Gran, granulocyte. **(D)** Representative histological images of the lung, spleen, liver, kidney, and heart of mice stained with H&E after BCCY-1 treatment. Bar = 50 μm.

Firstly, we evaluated the biosafety of BCCY-1 *in vivo*. Peptide BCCY-1 was intraperitoneally injected into C57BL6/J mice at the indicated dosage. The results showed that there was no significant difference in the evaluation index of the BCCY-1 treated group compared with the saline control group, including food intake (**Figure 1A**), body weight (**Figure 1B**), blood routine (**Figure 1C**) and histological analysis (**Figure 1D**). Interestingly, the percentage of monocytes in the peripheral blood was not altered after *i.p.* injection of BCCY-1 in mice (**Figure 1C**).

### *In Vivo* Biodistribution of BCCY-1

**Figure 2:**
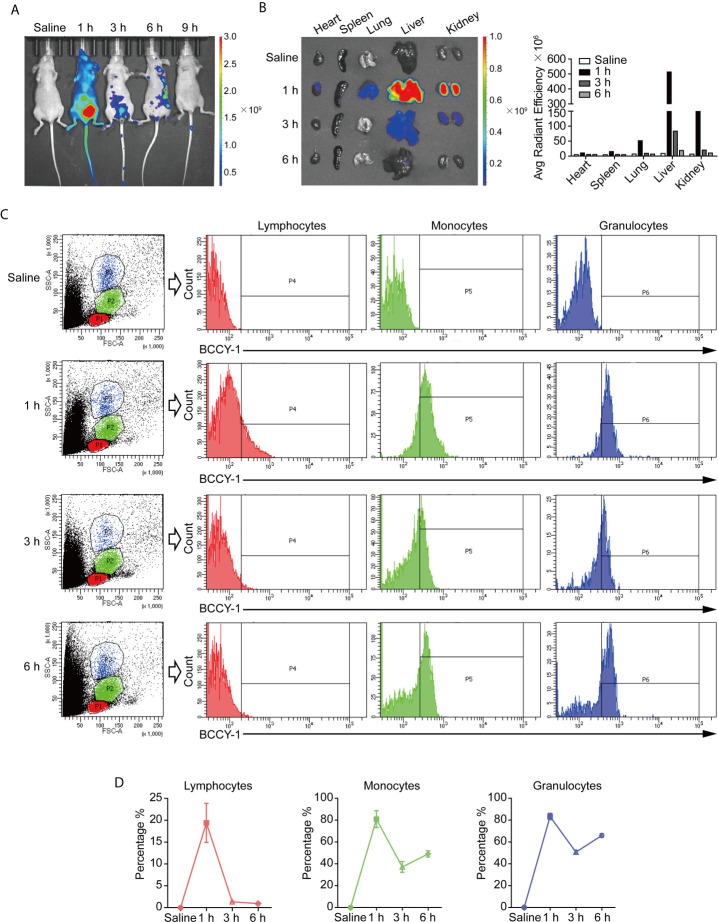
*In vivo* biodistribution analysis of BCCY-1. **(A)** *In vivo* imaging of mice and **(B)** *ex vivo* imaging of major organs at indicated hours after TAMRA-BCCY-1 treatment. Average radiant efficiency ([p/s/cm^2^/sr]/[μW/cm^2^]) was quantified at indicated time points. **(C, D)** The biodistribution of BCCY-1 in peripheral blood cells (lymphocytes, monocytes and granulocytes) was analyzed by flow cytometry at indicated hours. The percentage of TAMRA positive cells in the indicated cell population was quantified.

The biodistribution of BCCY-1 was investigated by an *in vivo* imaging system after intraperitoneal injection. As shown in **Figure 2A**, the strongest fluorescence signal was obtained at 1 h post injection. Furthermore, *ex vivo* imaging of organs showed the distribution of BCCY-1 in heart, spleen, lung, liver and kidney (**Figure 2B**). A significantly lower distribution of BCCY-1 in lymphocytes was observed, along with a significantly higher accumulation in monocytes and granulocytes. More importantly, the fluorescence in monocytes and granulocytes can last over 6 h (**Figures 2C, D**). These findings suggested that BCCY-1 showed good system circulation that might contribute to a higher uptake and the subsequent modulatory effects of BCCY-1 in monocytes.

### Peptide BCCY-1 Could Enhance Monocyte Recruitment *In Vivo*

**Figure 3:**
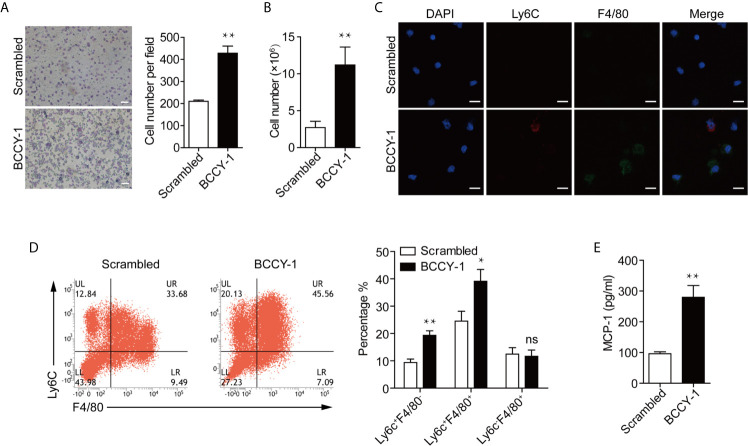
Effects of BCCY-1 on monocyte recruitment in *vivo*. Monocyte recruitment into the peritoneum induced by peptide BCCY-1 (10 mg/kg) post *i.p.* injection was analyzed. The scrambled version of BCCY-1 served as a negative control. **(A)** Wright-Giemsa stain of peritoneal lavage fluid after BCCY-1 or Scrambled peptide administration at 24 h. Bar=200 μm. The nucleated cell number per field is shown as a bar graph in the right panel. **p < 0.01. **(B)** Cell number of peritoneal lavage cells after BCCY-1 or Scrambled peptide administration at 48 h. **p < 0.01. **(C)** Representative confocal images of peritoneal lavage cells after the indicated treatment are shown. Nuclei were stained with DAPI (Blue: DAPI; Red: Ly6C; Green: F4/80). Bar=20 μm. **(D)** Monocyte recruitment at 48 h post BCCY-1 injection was analyzed by flow cytometry using the following markers: Ly6C (monocytes), F4/80 (macrophages). Quantification of monocyte/macrophage percentage is shown as a bar graph in the right panel. ns *p* > 0.05, *p < 0.05, **p < 0.01. **(E)** Peritoneal lavage fluids at 48 h after the indicated treatment were analyzed for MCP-1 production by ELISA. **p < 0.01.

Next, we sought to examine whether the chemokine induction activity of peptide BCCY-1, as we observed *in vitro* (9), could lead to a significant increase in monocyte recruitment *in vivo* following peptide administration. Peptide BCCY-1 or Scrambled peptide was given locally by the *i.p.* injection, and the peritoneal lavage was collected at 24 h post-injection. Peritoneal lavage cell smear was stained with Wright-Giemsa for histologic examination. Compared with Scrambled peptide-treated mice, animals stimulated by BCCY-1 yielded more nucleated cells in the peritoneal lavage fluid (**Figure 3A**). At 48 h post injection, the total number of nucleated cells in peritoneal lavage of BCCY-1-treated mice was significantly higher than that in Scrambled peptide-treated mice (**Figure 3B**). Distinct subsets of monocytes/macrophages could be distinguished by expression of the markers Ly6C and F4/80 (12). Interestingly, there was a significant increase in percentages of monocytes in the BCCY-1 treated peritoneal lavage gated as Ly6C^+^F4/80^-^ monocytes and Ly6C^+^F4/80^+^ monocytes/macrophages, respectively (**Figures 3C, D**). Moreover, the percentage of Ly6C^+^ monocytes in the peritoneal lavage fluid, but not in the peripheral blood, was increased upon BCCY-1 treatment **(Supplementary Figure 4**). In addition, BCCY-1 induced monocyte recruitment was associated with the increased level of the monocyte chemoattractant MCP-1 in the peritoneal lavage fluid (**Figure 3E**).

### Peptide BCCY-1 Demonstrates Anti-Infective Effects *In Vivo*

**Figure 4:**
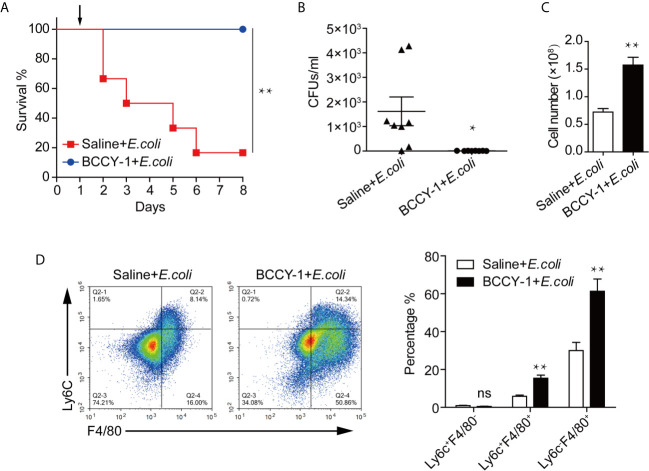
Efficacy of BCCY-1 in bacterial infection models. **(A)** C57BL6/J mice were infected by *i.p.* injection with 1×10^8^ CFUs/mouse of *E.coli*. BCCY-1 (10 mg/kg/day) or saline was administered by *i.p.* injection 48 h before *E.coli* challenge. Survival of mice was recorded. The day of challenge was designated as day 1 (black arrow). n=6. **(B)** Mice were treated *i.p.* with BCCY-1 (10 mg/kg/day) 48 h hours before *E.coli* challenge. The bacterial load in the peritoneal lavage fluid after 24 h of infection was shown by CFU counts. n=8. *p < 0.05. **(C)** Cell number of nucleated cells in peritoneal lavage fluid of each group. n=8. **p < 0.01. **(D)** Monocyte recruitment into the peritoneum after infection in each group was analyzed by flow cytometry. Quantification of monocyte/macrophage percentage is shown as a bar graph in the right panel. n=5. ns *p* > 0.05, **p < 0.01.

The anti-infective potential of BCCY-1 was determined using a mouse model of aggressive bacterial infection as previously described (13). Peptide BCCY-1 or saline was given 48 h prior bacterial challenge. Compared with saline-treated control, BCCY-1 significantly decreased the mortality (**Figure 4A**) and the bacterial CFU counts (**Figure 4B**) in infected mice. The total number of nucleated cells (**Figure 4C**) and percentages of Ly6C^+^F4/80^+^ monocytes/macrophages and Ly6C^-^F4/80^+^ macrophages in the peritoneal lavage fluid (**Figure 4D**) of BCCY-1-treated infected mice was significantly higher than that in saline-treated mice. However, the MCP-1 level in the peritoneal lavage fluid of group “BCCY-1+*E.coli* “ was significantly lower than that in group “Saline+*E.coli*” (**Supplementary Figure 5**). These data demonstrated that BCCY-1 could efficiently control infection without an increase in potentially harmful inflammation.

### The *In Vivo* Anti-Infective Effect of BCCY-1 Depends on Enhancing Monocyte Recruitment

**Figure 5:**
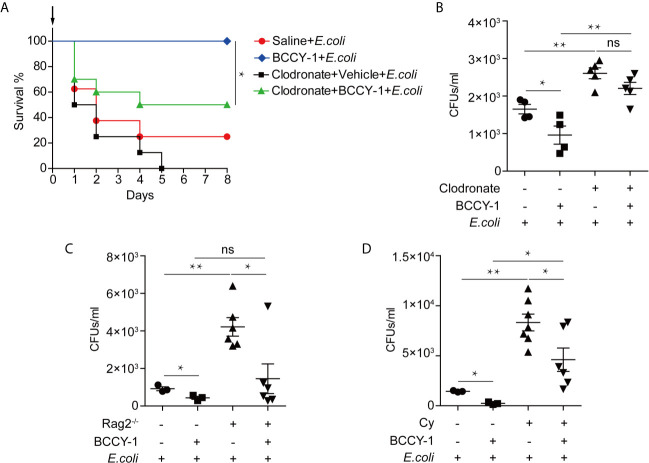
Effects of BCCY-1 *in* specific cell-depletion mouse models. To deplete monocytes/macrophages, C57BL6/J mice were treated once with clodronate liposomes. Two days after liposome treatment, BCCY-1 (10 mg/kg/day, for 2 days) or saline was administered by *i.p.* injection. After 48 h administration, mice were infected with *E.coli via i.p.* injection. **(A)** The day of challenge was designated as day 0 (black arrow). Survival of mice (n=8-10) was measured and **(B)** bacterial CFU counts were determined in the peritoneal lavage at 24 h after infection. n=4-5, ns *p* > 0.05, *p < 0.05, **p < 0.01. **(C)** The bacterial CFU counts were determined in the peritoneal lavage fluid of the lymphopenia models at 24 h after infection. n=3-6, ns *p* > 0.05, *p < 0.05, **p < 0.01. **(D)** The bacterial CFU counts were determined in the peritoneal lavage of the neutropenic models at 24 h after infection. n=3-7, Cy: Cyclophosphamide, *p < 0.05, **p < 0.01.

**Figure 6:**
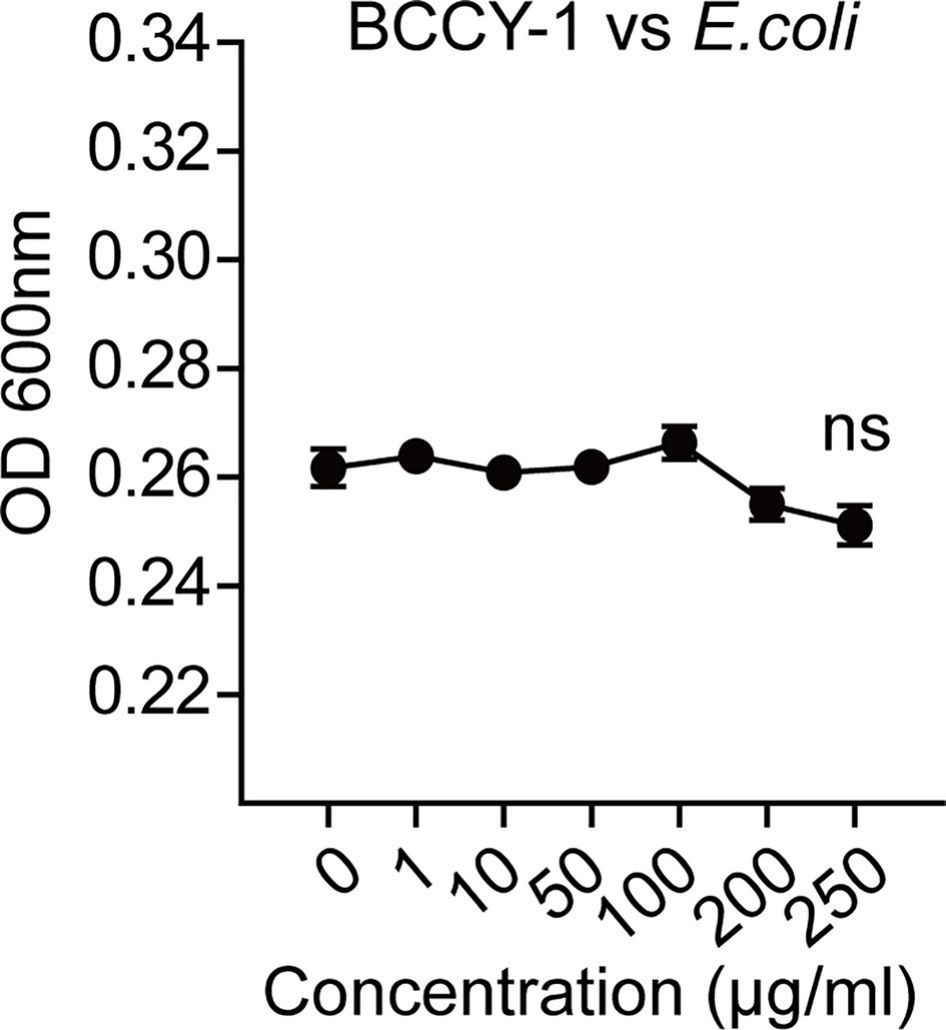
BCCY-1 lacks directly antimicrobial effect. The direct effect of BCCY-1 on the growth of *E.coli.* ns *p* > 0.05.

To further determine the key effector cell in BCCY-1-mediated protection, we administered peptide BCCY-1 in the infection models with deficient in monocytes/macrophages, T/B lymphocytes and neutrophils, respectively. We found that depletion of monocytes/macrophages using clodronate liposomes abrogated the protective activity of BCCY-1 (**Figures 5A, B**), while protection was still observed in mice deficient in T/B lymphocytes (**Figure 5C**) and neutrophils (**Figure 5D**), confirming the pivotal role for monocytes/macrophages in BCCY-1-mediated protection. Additionally, no significant alteration of bacterial growth was detected in the present of BCCY-1 using the *in vitro* antimicrobial assay (**Figure 6**).

## Discussion

Although the clinical benefits to the infants of breastfeeding in host defense against infection are well documented, there are still intriguing mysteries of how human milk directly contributes to the infant’s immune function and protecting against infections (2). Numerous milk-derived multifunctional compounds including secretory type A immunoglobulin (sIgA), lactoferrin, and oligosaccharides are identified with potential applications as clinical therapies in infectious disease (14). The interests on bioactive peptides derived from milk protein are increasing because of their very diverse activities, which not only provide a source of essential amino acids beneficial for the rapidly growing infants, but also exert physiological functions and contribute to neonatal health through many different ways (15). For example, a novel peptide HLR1r, derived from the human milk protein lactoferrin is directly antibacterial and demonstrates pronounced anti-infectious effect both *in vitro* and *in vivo* (16). Although the immunostimulatory effects of diverse milk protein-derived peptides has been reported (17), the potential function of these peptides in anti-infection remains largely unknown and the underlying mechanism is still unclear.

In the previous study, we have found that a novel human β-casein derived peptide BCCY-1, which was identified in our previous research on the peptidomics of human breast milk (8), can enhance the chemokine production and cell migration of monocytes *in vitro*. And this immunomodulatory activity is dependent on its ability to activate the NF-κB and MAPK signaling pathways (9). As we known, recruitment of phagocytic innate immune cells, such as monocytes and neutrophils, is essential for effective control and clearance of infections (18). During the course of infection, circulating monocytes traffic through the bloodstream and migrate into the site of infection, where they differentiate into macrophages or dendritic cells to restrict further growth and invasion of pathogens (19). Chemokines play important roles in immune cell recruitment *in vivo*. It has been demonstrated that the *ex vivo* chemokine-induction property is a promising method for the development of immunomodulatory peptides with anti-infective activity. The human host defense peptide (HDP) LL-37 can promote production of chemokines (e.g. CXCL1 and IL-8) and leukocyte recruitment contributing to the resolution of infections (20). The synthetic innate defense regulator (IDR) peptide, IDR-HH2, IDR-1002 and IDR-1018, can increase the protection from bacterial infections through the induction of chemokines and the recruitment of neutrophils to the site of infection (17, 21). Therefore, our previous *in vitro* study suggests an anti-infective potential of peptide BCCY-1 which needs to be further investigated.

In the present study, the *in vivo* characteristics and anti-infective effect of peptide BCCY-1 were evaluated. We found that peptide BCCY-1 exhibited negligible toxicity and favorable biosafety *in vivo*. There were no noticeable abnormal food intake and weight change observed among the mice intraperitoneally injected with peptide BCCY-1. The major organs showed no obvious injury and the hematology parameters exhibit no significant variation after treatment. The biodistribution analysis revealed that BCCY-1 were mainly cleared by the liver and kidney. Moreover, BCCY-1 showed a higher affinity and longer retention in monocytes and granulocytes than in lymphocytes, suggesting that BCCY-1 has the innate immune cell accumulation abilities significantly.

Host defense against bacterial infection involves a complex network of cells and molecules of the innate and adaptive immunity. For example, upon *L. monocytogenes* infection, splenic dendritic cells (DCs) could coordinate natural killer (NK) cell and monocyte recruitment to the infected spleen. Recruited NK cells, in proximity to recruited Ly6C^hi^ monocytes, produce interferon−γ (IFN-γ) and thus drive monocyte differentiation into TNF- and iNOS-producing DCs at the site of infection (22). The antimicrobial role of Ly6C^hi^ monocytes in mouse have been reported (23). CCR2 deficiency impairs recruitment of Ly6C^hi^ monocytes to infected sites and worsens infection with *L. monocytogenes* (19). In agreement with this finding, we found that the percentage of Ly6C^+^ monocyte recruitment into the peritoneum, but not in the peripheral blood, was increased upon BCCY-1 treatment, further confirming the potential role of BCCY-1 against infection. Furthermore, recruited monocytes are thought to be the major producers of key cytokines that trigger subsequent responses. For example, Ly6C^hi^ monocytes are required for IFN-γ production by γδ T cells and NK cells, allowing for enhanced control of *L. pneumophila* infection (23).

Consistent with our *in vitro* results that BCCY-1 induced the monocyte chemotactic protein 1 (MCP-1) production and migration of monocytes (9), BCCY-1 strikingly enhanced the MCP-1 production and monocyte recruitment *in vivo*. The scrambled version of peptide BCCY-1 exerted no such effect. Importantly, treatment with BCCY-1 protected mice from pathogen-caused lethal infections by promoting recruitment of monocytes/macrophages to the site of infection. Interestingly, BCCY-1 has no direct antimicrobial effect *in vitro*. MCP-1 is one of the key chemoattractants that responsible for the migration and infiltration of monocytes/macrophages, but also attracts T lymphocytes and neutrophils (24). Our *in vivo* data of the specific cell-deficient mouse model revealed that all three cell types including monocytes/macrophages, T/B lymphocytes and neutrophils were involved in the host defense against bacterial infection. However, monocytes/macrophages were the key effector cells in the BCCY-1-mediated protection. Nevertheless, the peritoneal lavage CFU counts in mice treated with BCCY-1 were significantly increased upon neutrophil depletion, suggesting an important role of neutrophils in the resolution of infection. It is worth noting that administration of MCP-1 could also protect animals from the lethal effects of Lipopolysaccharides (LPS) (25), *Salmonella typhimurium* and *Pseudomonas aeruginosa* by itself (26). The properties of BCCY-1 are reminiscent of the immunomodulatory activities of peptide IDR-1, which represents a “resistance-free” concept in treating infections that complement the use of antibiotics as the peptide is not directly antimicrobial (11).

The innate immune response is the first line of host defense against infection. However, reduced cytokine production, poor monocyte function and immune responses are the features of newborn infants’ immature immune system (9), resulting in a diminished infection-fighting capacity of newborns (27). In addition, impaired immune function and eventual immunosuppression have been found in the early stage of infection (28). Thus, improving host immune function is crucial to prevent the onset of infections. In this context, peptide BCCY-1 derived from human milk protein casein could selectively enhance the recruitment and activity of monocytes/macrophages, which are important for host immune responses to clear infection. Our findings clarify the favorable nature of human milk bioactive peptides in anti-infective protection.

**Figure 7:**
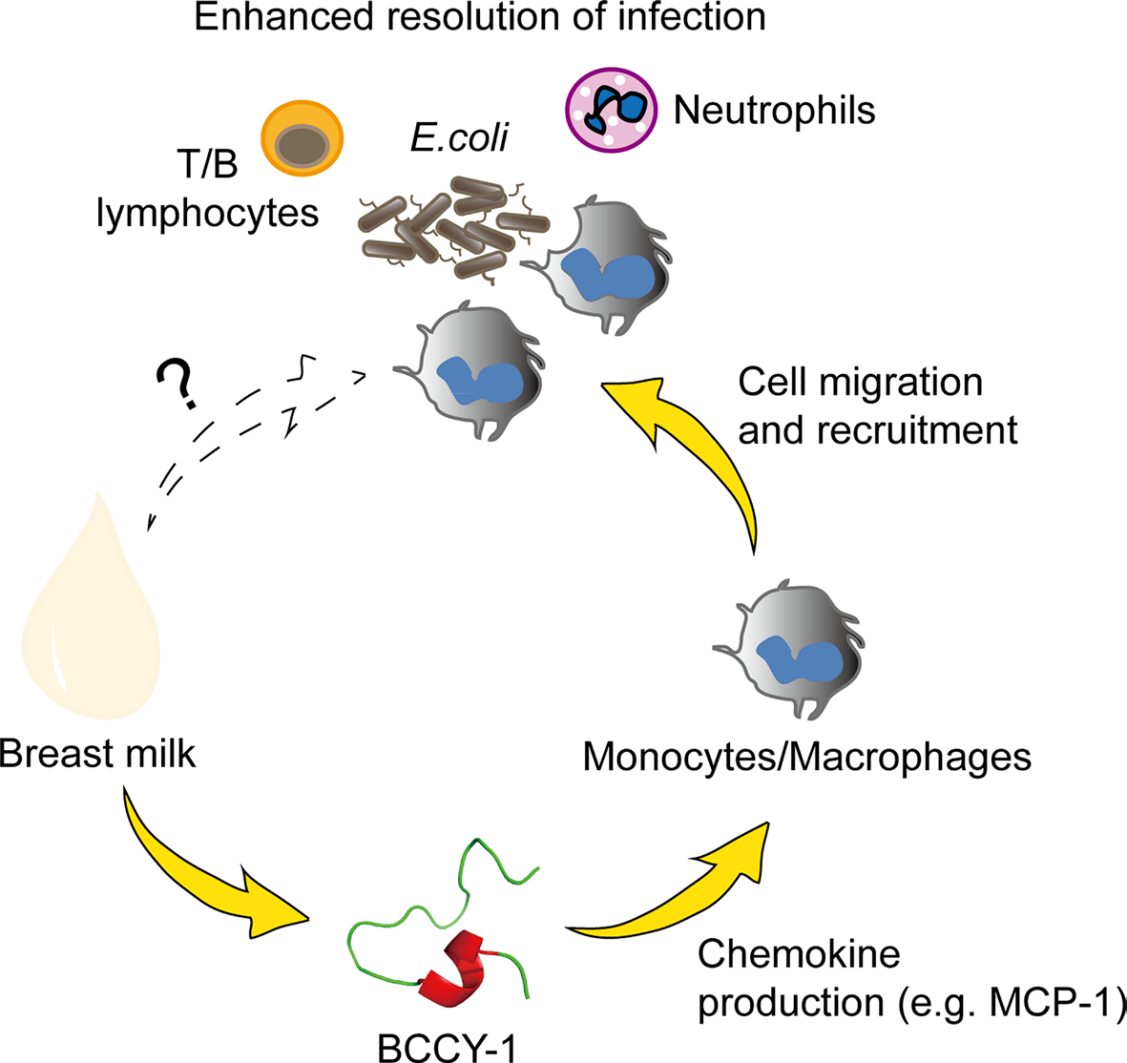
Proposed anti-infective action of peptide BCCY-1. Monocytes/macrophages, T/B lymphocytes and neutrophils are all involved in the host defense against bacterial infection. The human β-casein-derived peptide BCCY-1 enhances the production of chemokines, such as MCP-1. As a result, monocytes/macrophages are recruited to the infection site and assist in resolving bacterial infections (e.g. *E.coli*).

In conclusion, we demonstrated that peptide BCCY-1 promoted the resolution of infection by selectively enhancing monocyte recruitment without direct antimicrobial activity (**Figure 7**). These findings provide novel insight into the mechanism by which the milk protein derived peptides may contribute to the health benefits of breastfeeding, particularly on “immunopromoting” and fighting against infection. Moreover, the immunomodulatory properties of peptide BCCY-1 represents a novel approach to anti-infective strategy which may has competitive advantages over directly microbicidal agents. To the best of our knowledge, this is the first report of the anti-infective effects of the casein derived peptide BCCY-1 *in vivo*.

## Data Availability Statement

The original contributions presented in the study are included in the article/**Supplementary Material**. Further inquiries can be directed to the corresponding author.

## Ethics Statement

The animal study was reviewed and approved by Institutional Review Board at Women’s Hospital of Nanjing Medical University, Nanjing Maternity and Child Health Care Hospital ([2017]15).

## Author Contributions

Collected and analyzed the data: JC and YC. Methodology and validation: XC and XW. Review and editing: LY, CJ, and YC. Designed the study, interpretation of data, and wrote the manuscript: YC. All authors contributed to the article and approved the submitted version.

## Funding

This study was supported by National Natural Science Foundation of China (81701491); The Natural Science Foundation of Jiangsu Province, China (BK20170152); Science and Technology Development Foundation of Nanjing Medical University (2016NJMUZD060, 2017NJMU017).

## Conflict of Interest

The authors declare that the research was conducted in the absence of any commercial or financial relationships that could be construed as a potential conflict of interest.

## Supplementary Material

Supplementary Figure 1 The FACS gating strategy used for the analysis of the biodistribution of TAMRA-BCCY-1 in the peripheral blood cells. Peripheral blood cells can be roughly differentiated into 3 main populations on FSC/SSC (P1- Lymphocytes, P2- Monocytes and P3- Granulocytes). The percentage of TAMRA-BCCY-1 positive cells in each population was analyzed.

Supplementary Figure 2 The FACS gating strategy used for the analysis of the monocyte/macrophage populations in the peritoneal lavage fluid. The distinct subsets of monocytes/macrophages could be distinguished by expression of the markers Ly6C and F4/80. Monocytes: Ly6C^+^F4/80^-^, monocytes/macrophages: Ly6C^+^F4/80^+^, macrophages: Ly6C^-^F4/80^+^.

Supplementary Figure 3 Flow charts showing the timing of experimental treatments and infection. **(A)** The systemic infection model, **(B)** The clodronate model, **(C)** The neutropenic model, Cy: Cyclophosphamide, **(D)** the lymphopenia model.

Supplementary Figure 4 The percentage of Ly6C^+^ monocytes after BCCY-1 treatment. **(A)** In the peripheral blood. Quantification of Ly6C^+^ monocyte percentage is shown as a bar graph in the right panel. ns *p* > 0.05. **(B)** In the peritoneal lavage fluid. Quantification of Ly6C^+^ monocyte percentage is shown as a bar graph in the right panel. ***p* < 0.01. The scrambled version of BCCY-1 served as the control.

Supplementary Figure 5 The MCP-1 level in the peritoneal lavage fluid of *E. coli*-infected mouse after Saline or BCCY-1 treatment. Peritoneal lavage fluids of the indicated treatment after 24 h of infection were analyzed for MCP-1 production by ELISA. ***p* < 0.01.
