# Supplementary figures and images for: A Novel Anti-Infective Peptide BCCY-1 With Immunomodulatory Activities
Source: Front Immunol. 2021 Jul 22;12:713960. doi: 10.3389/fimmu.2021.713960 (PMC8339908; doi:10.3389/fimmu.2021.713960)

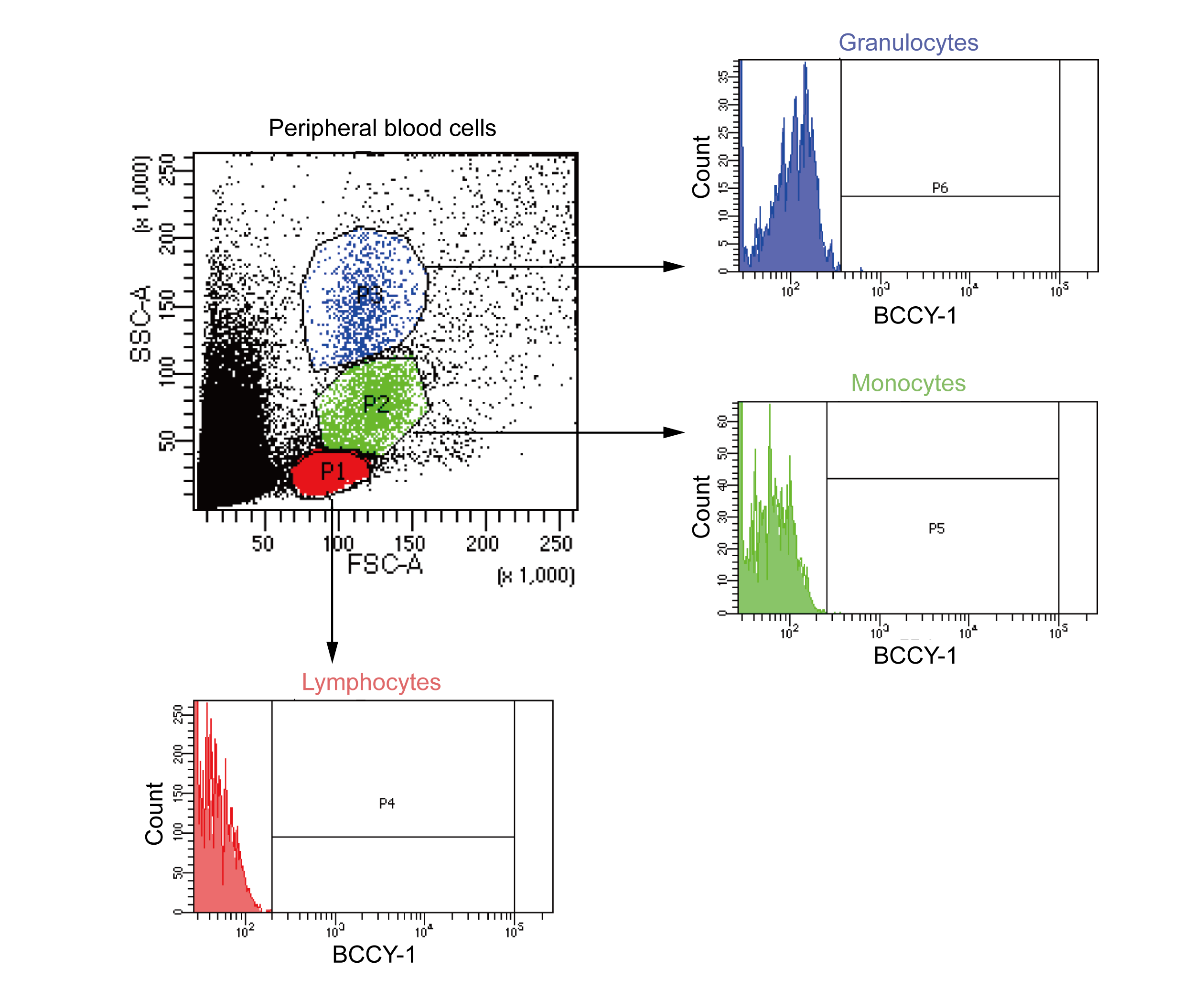

Supplement: Supplementary Figure 1 — The FACS gating strategy used for the analysis of the biodistribution of TAMRA-BCCY-1 in the peripheral blood cells. Peripheral blood cells can be roughly differentiated into 3 main populations on FSC/SSC (P1- Lymphocytes, P2- Monocytes and P3- Granulocytes). The percentage of TAMRA-BCCY-1 positive cells in each population was analyzed. [file Image_1.tif]

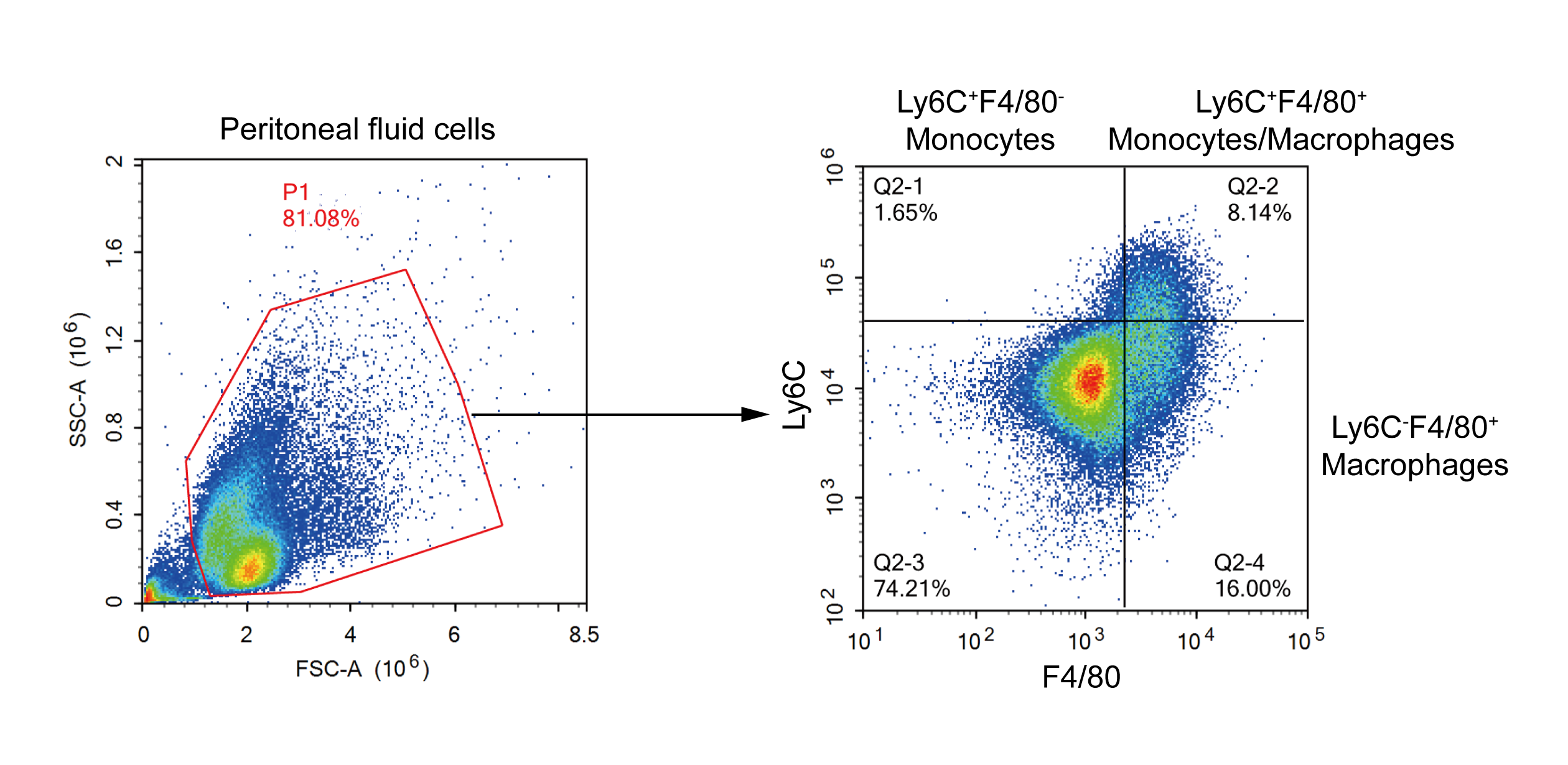

Supplement: Supplementary Figure 2 — The FACS gating strategy used for the analysis of the monocyte/macrophage populations in the peritoneal lavage fluid. The distinct subsets of monocytes/macrophages could be distinguished by expression of the markers Ly6C and F4/80. Monocytes: Ly6C+F4/80-, monocytes/macrophages: Ly6C+F4/80+, macrophages: Ly6C-F4/80+. [file Image_2.tif]

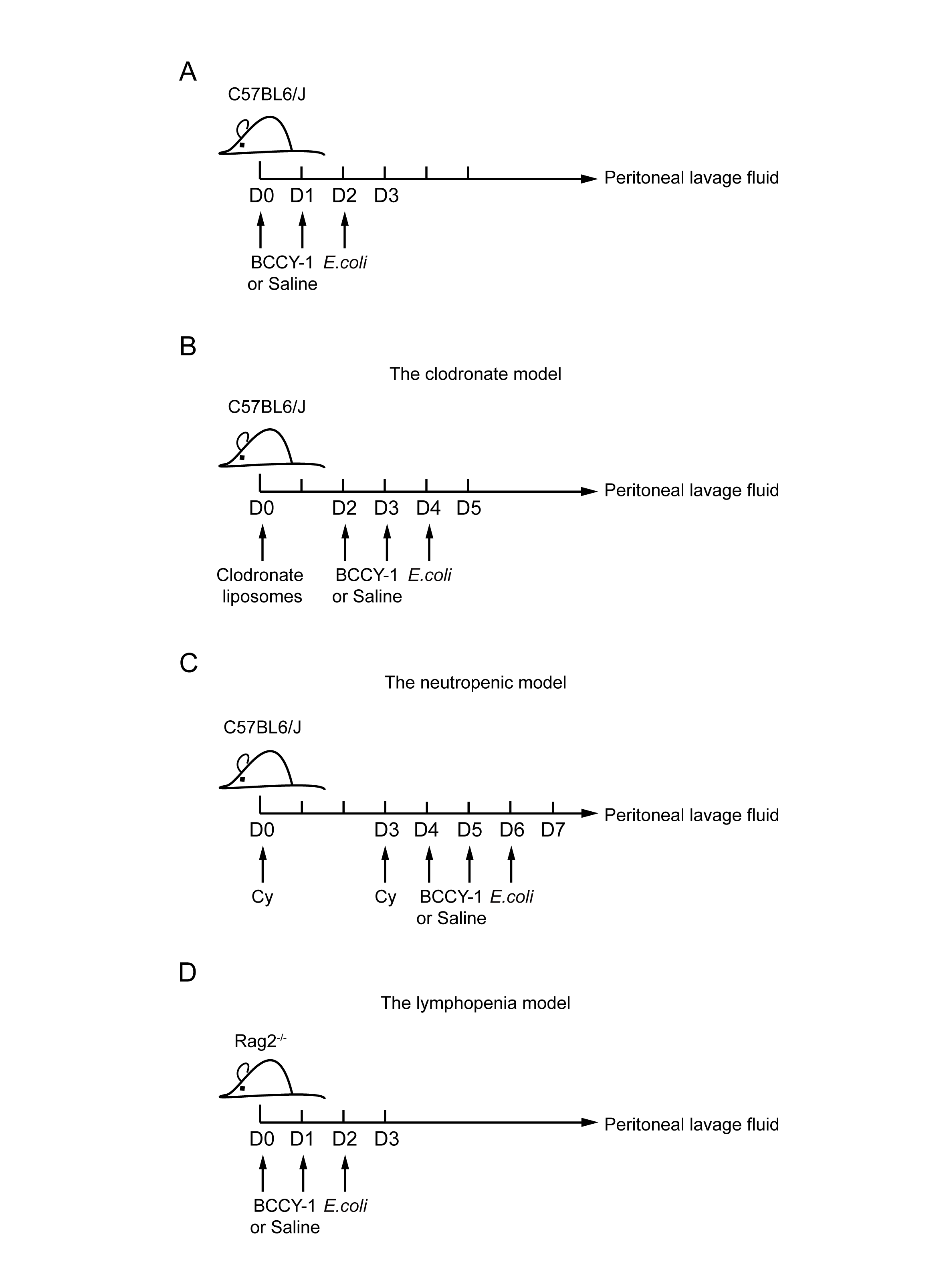

Supplement: Supplementary Figure 3 — Flow charts showing the timing of experimental treatments and infection. (A) The systemic infection model, (B) The clodronate model, (C) The neutropenic model, Cy: Cyclophosphamide, (D) the lymphopenia model. [file Image_3.tif]

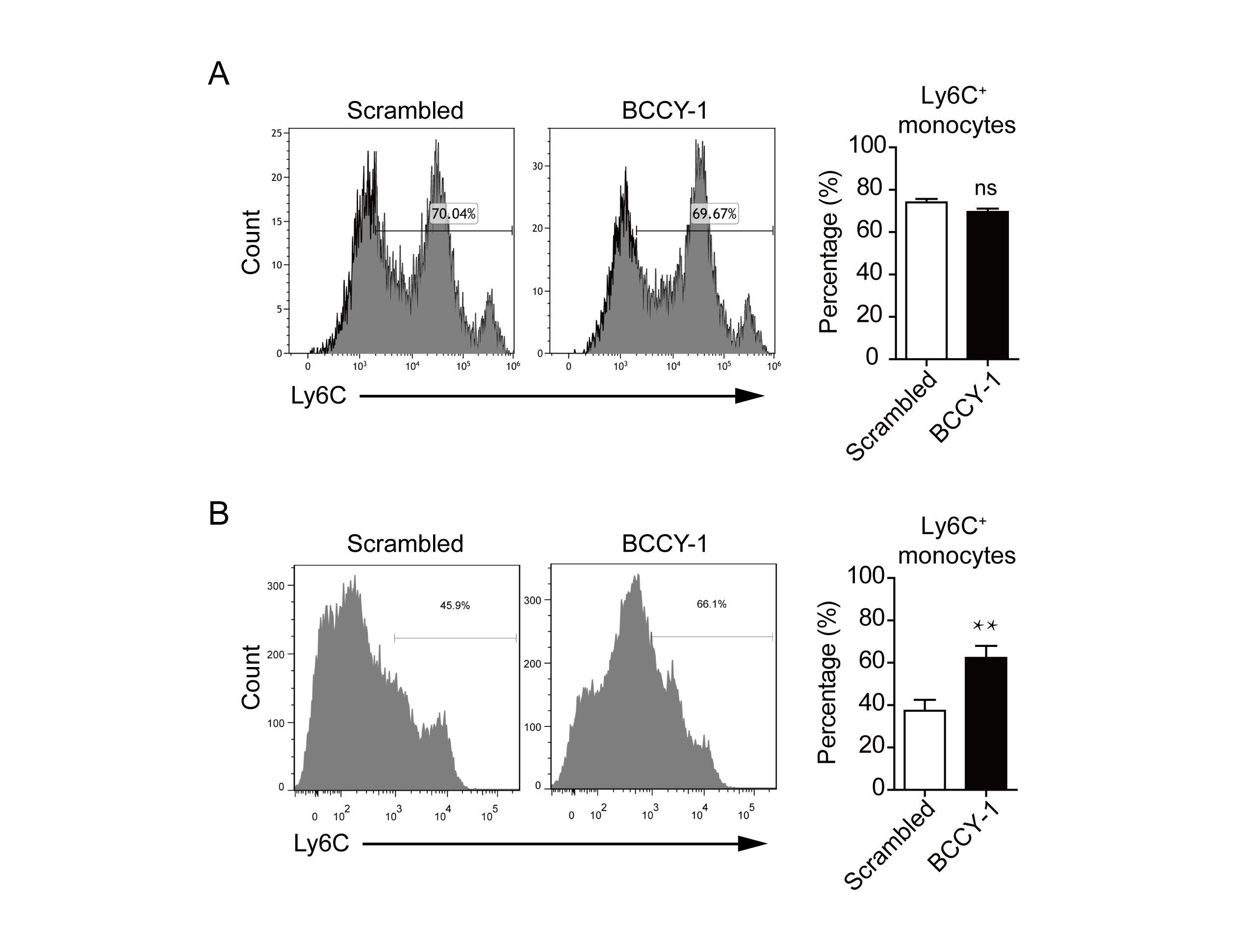

Supplement: Supplementary Figure 4 — The percentage of Ly6C+ monocytes after BCCY-1 treatment. (A) In the peripheral blood. Quantification of Ly6C+ monocyte percentage is shown as a bar graph in the right panel. ns p > 0.05. (B) In the peritoneal lavage fluid. Quantification of Ly6C+ monocyte percentage is shown as a bar graph in the right panel. **p < 0.01. The scrambled version of BCCY-1 served as the control. [file Image_4.tif]

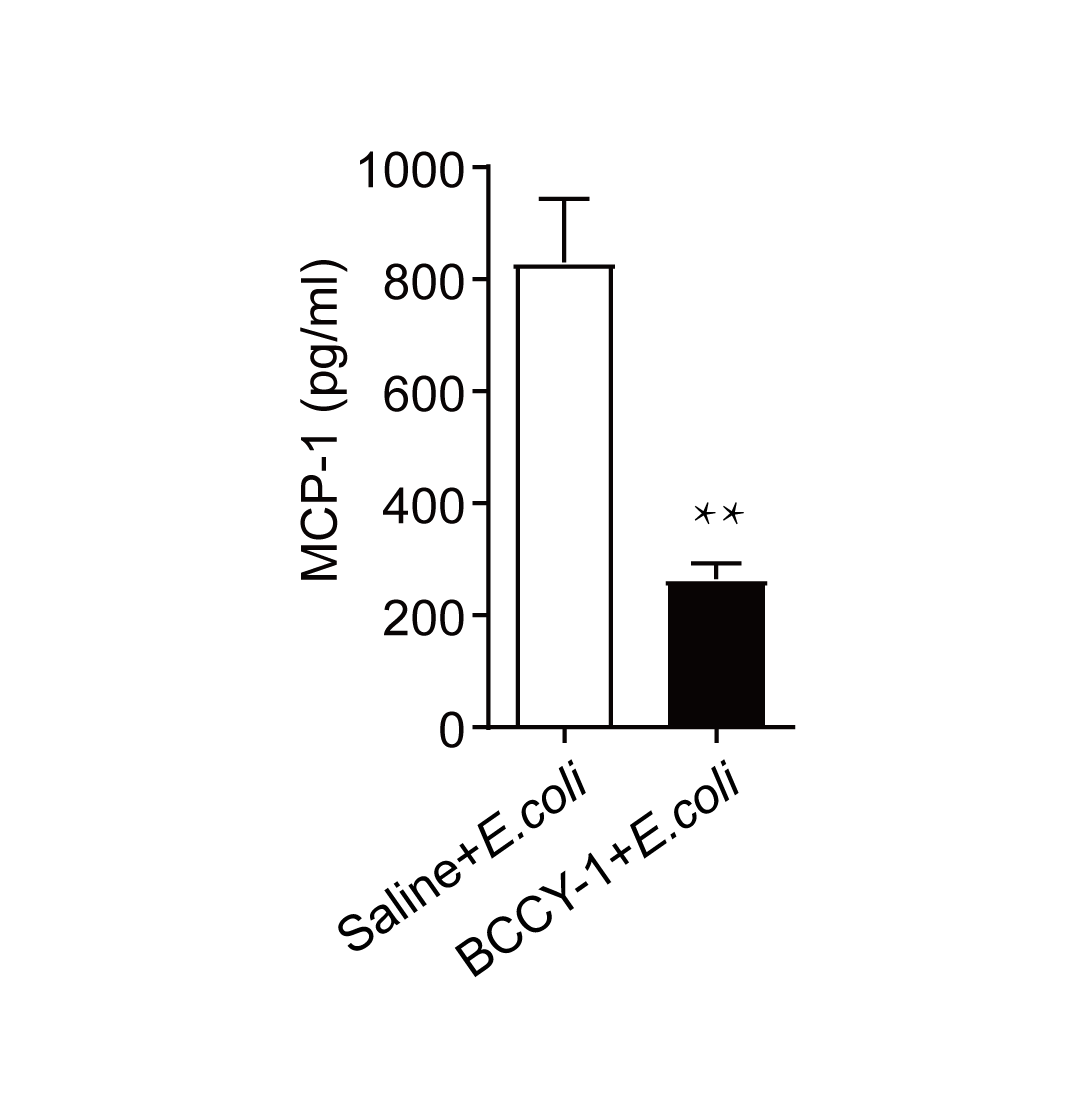

Supplement: Supplementary Figure 5 — The MCP-1 level in the peritoneal lavage fluid of E. coli-infected mouse after Saline or BCCY-1 treatment. Peritoneal lavage fluids of the indicated treatment after 24 h of infection were analyzed for MCP-1 production by ELISA. **p < 0.01. [file Image_5.tif]
